# The target sign: a significant CT sign for predicting small-bowel ischemia and necrosis

**DOI:** 10.1007/s11547-024-01793-z

**Published:** 2024-02-14

**Authors:** Bo Li, Zhifeng Wu, Jinjun Wang

**Affiliations:** 1 Eighth Hospital of Shanxi Medical University, Yuncheng Central Hospital, No.3690 Hedong East Street, Yanhu District, Yuncheng City, Shanxi Province China; 2 Third Hospital of Shanxi Medical University, Shanxi Bethune Hospital, No. 99 Longcheng Street, Taiyuan City, Shanxi Province China

**Keywords:** CT scanning, Ischemia and necrosis, Small-bowel obstruction

## Abstract

**Objective:**

To investigate the correlation between changes in the thickness and density of diseased small-bowel wall and small-bowel ischemia and necrosis (SBN) on CT imaging when small-bowel obstruction (SBO) occurs.

**Methods:**

We retrospectively analyzed 186 patients with SBO in our hospital from March 2020 to June 2023. The patients were divided into simple SBO (control group) and SBN (case group) groups. We used logistic regression analysis, the chi-square test, and Fisher's exact test to analyze the correlation between the changes in the thickness and density of the diseased intestinal wall and the SBN. A receiver operating characteristic (ROC) curve was used to calculate the accuracy of the multivariate analysis.

**Results:**

Of the 186 patients with SBO, 98 (52.7%) had simple SBO, 88 (47.3%) had SBN, and the rate of SBN was 47.3% (88/186). Multivariate regression analysis revealed that six CT findings were significantly correlated with SBN (*p* < 0.05), namely, thickening of the diseased intestinal wall with the target sign (OR = 21.615), thinning of the diseased intestinal wall (OR = 48.106), increase in the diseased intestinal wall density (OR = 13.696), mesenteric effusion (OR = 21.635), decrease in the diseased intestinal wall enhancement on enhanced scanning (OR = 41.662), and increase in the diseased intestinal wall enhancement on enhanced scanning (OR = 15.488). The AUC of the multivariate analysis reached 0.987 (95% CI 0.974–0.999). Specifically, the target sign was easily recognizable on CT images and was a significant CT finding for predicting SBN.

**Conclusion:**

We identified 6 CT findings that were significantly associated with SBN, and may be helpful for clinical treatment.

## Introduction

Small-bowel obstruction (SBO) is one of the most common types of acute abdomen in clinical practice, and accounts for approximately 12–16% of all emergent surgical procedures [1]. SBO can be divided into simple SBO and small-bowel ischemia and necrosis (SBN), and most simple SBO can be relieved or treated through active conservative treatment such as gastrointestinal decompression [2]. SBN is a serious complication of SBO. Patients with SBN require urgent surgical treatment; otherwise, they may develop secondary systemic infections, sepsis, or even death [3]. Therefore, accurately distinguishing the type of SBO and whether there is an SBN before surgery is highly important for treatment decision-making.

Compared to other examinations, abdominal CT scanning is an important means of diagnosing SBO and SBN, with higher sensitivity (73–100%) and specificity (61–100%) [4, 5]. Due to the inability of abdominal X-ray images to reveal changes in the small-bowel wall and mesentery, diagnosing SBN using abdominal X-ray examination is difficult. SBN may be present only when pneumoperitoneum is caused by small-bowel perforation [6]. Full abdominal CT can clearly display changes in the thickness and density of the small-bowel wall, as well as reveal structures such as the small-bowel mesentery and blood vessels, providing significant clues for predicting SBN. Previous studies have shown that CT manifestations of SBN after SBO include thickening of the small-bowel wall, decreased enhancement, small-bowel mesenteric edema, and peritonitis [1, 2]. Abdominal MRI scanning has limited diagnostic value for SBN due to respiratory artifacts [4].

Many previous studies have focused on using clinical signs and laboratory tests to predict SBN, while the role of CT findings and signs has been underestimated [7–9]. In other studies, multiple CT signs and clinical laboratory indicators have been used to jointly predict SBN, and predictive models have been developed to predict SBN [3, 10, 11]. However, few studies have focused on exploring the changes in the thickness and density of diseased intestinal wall itself during SBN. Compared to other laboratory tests or other CT signs, such as the whirl sign and intestinal wall gas accumulation, changes in the density or thickness of the diseased intestinal wall during SBN may be more common, because SBN is mostly caused by compression and strangulation of small-bowel drainage vessels or thrombus blockage, which mostly causes changes in the thickness and density of the ischemic or necrotic small-bowel wall on CT images [12, 13].

Therefore, in this study, our aim was to identify CT findings or signs of possible changes in the thickness and density of the diseased intestinal wall that were significantly related to SBN.

## Materials and methods

### Patient selection

Our research was approved by the Ethics Review Committee of our hospital and informed consent was obtained from all patients. The ethics committee approval number of our study was YXLL2023036. We retrospectively analyzed the CT images of consecutive patients with SBO admitted to our hospital between March 2020 and June 2023. The eligibility criteria included the following: (1) surgical treatment was ultimately performed at our hospital; and (2) all patients underwent preoperative plain and enhanced CT scans of the entire abdomen. The exclusion criteria for patients were as follows: (1) had paralytic intestinal obstruction caused by abdominal inflammation, appendicitis, cholecystitis, etc.; (2) had large intestine obstruction; (3) had small-bowel perforation accompanied by SBO caused by small-bowel foreign bodies, sharp instrument thrusts, etc.; (4) had only a plain CT scan performed without an enhanced CT scan; and (5) had small-bowel or abdominal tumors. SBOs caused by small-bowel or abdominal tumors often present with a chronic course [14], so these patients with SBO were not included in our research.

In our research, the diagnostic criterion for SBO according to CT were diffuse or partial dilation of the intestinal lumen for various reasons, with the inner diameter of the dilated intestinal lumen exceeding 3.0 cm. Except for paralytic intestinal obstruction, most SBO can be observed within the transition zone, and the distal intestinal lumen after the transition zone often collapses or becomes normal. In some patients with SBO, gas‒liquid levels can be observed [4].

### The CT scanning technique

In this study, all patients underwent plain and enhanced abdominal CT scans using a Philips Brilliance 64 CT scanner or a GE Revolution 256 CT scanner at our hospital. The CT scanning protocol was as follows: (1) Plain scanning: the layer thickness was 0.625 mm, and the reconstruction layer thickness was 1.25 mm, with an interval of 1.25 mm.The scanning range was from the diaphragm to the pubic symphysis, including part of the lower lobes of both lungs. (2) Enhanced scanning, which included an intravenous injection of iodine contrast agent at a dose of 1.5–2 mL/kg and a flow rate of 3 mL/s, was conducted. The arterial phase scanning time was approximately 25–30 s after injection of the contrast agent, and the portal vein phase scanning time was approximately 70–80 s, with a delayed scanning time of approximately 3 min. The layer thickness was 0.625 mm, the reconstruction layer thickness was 1.25 mm, and the interval was 1.25 mm. Coronal and sagittal reconstruction with a thickness of 3 mm was performed; (3) None of the patients received oral contrast agents. The observation of the degree of small-bowel wall enhancement after enhanced scanning mainly relies on portal vein phase scanning.

### Grouping and comparison

According to the inclusion and exclusion criteria, a radiologist with 17 years of experience in imaging diagnosis continuously selected patients with SBO from our hospital's radiology database, and extracted the following information from each patient's medical records: name, age, sex, whether surgical treatment was ultimately performed, intraoperative course records, and postoperative pathological examination results. Based on the intraoperative manifestations and postoperative pathological examinations, we divided the patients with SBO into an SBN group or the case group, and a simple SBO group or the control group. Reversible small-bowel ischemia is characterized by black or purple small-bowel walls during surgery, with impaired blood supply. After the obstruction is relieved or the diseased small-bowel is placed in warm saline, the color of the small-bowel can be restored. However, due to the possibility of reversible small-bowel ischemia progressing quickly to small-bowel necrosis without surgical intervention, in our study, patients with reversible small-bowel ischemia during surgery were classified into the SBN group.

### Image analysis

Two radiologists with 20 and 17 years of experience in gastrointestinal CT diagnosis independently and blindly recorded and analyzed the CT imaging data of all patients with SBO. The two radiologists were not aware of the surgical records, postoperative pathological results, or patient grouping, but were aware that this was only a retrospective study on the correlation between SBN and the changes in the small-bowel wall thickness and density. The consistency assessment between the two observers was completed using Cohen's kappa statistic by the first radiologist responsible for grouping.

Two radiologists recorded and analyzed the following CT findings and signs:

1. The transitional zone or approximate transitional zone of the SBO was searched to determine whether the SBO was a paralytic intestinal obstruction. Patients with paralytic intestinal obstruction were excluded.
2. On CT plain scanning, it was determined whether there were diffuse or partial thickness changes in the thickness of the diseased intestinal wall, and whether there were target signs. In this study, we defined a diseased small-bowel wall thickness ≥ 6 mm as a thickening in the small-bowel wall, and a diseased small-bowel wall thickness ≤ 2 mm as a thinning [4, 15]. Due to the significant influence of small-bowel dilation on the thickness of the intestinal wall, when it was difficult to accurately measure the thickness of the significantly dilated diseased intestinal wall, we compared the diseased intestinal wall thickness with the surrounding normal intestinal wall thickness to evaluate the changes in thickness of the diseased intestinal wall. Sudden thinning or thickening of the diseased intestinal wall was also considered abnormal.
3. It was determined whether there were diffuse or partial density changes in the diseased intestinal wall on CT plain scanning, and the degree of enhancement in the diseased intestinal wall after CT enhanced scanning. On CT plain scanning, if the CT value of the intestinal wall was lower than 20 HU, it was defined as an decrease in density, while the CT value of the intestinal wall greater than 50 HU was considered an increase in density [4, 15, 16]. If the intestinal wall became significantly thinner, the region of interest (ROI) was too small, and if the CT value of the intestinal wall could not be accurately measured, we determined the density changes in the diseased intestinal wall by comparing the density of the surrounding normal intestinal tissues. The change in CT value of the intestinal wall after enhanced scanning was less than 10 HU, which was considered a decrease in intestinal wall enhancement. A change in the CT value of the intestinal wall greater than 40 HU was considered an increase in intestinal wall enhancement [4, 16, 17]. When the changes in CT value of the intestinal wall could not be accurately measured, we still determined the degree of enhancement of the diseased intestinal wall by comparing the changes in density of the surrounding normal intestinal tissues.

### Statistical analysis

In our study, there were a total of 5 variables, all of which were categorical variables, namely, changes in the diseased intestinal wall thickness on plain scan (X_1_), changes in the diseased intestinal wall density on plain scanning (X_2_), mesenteric edema and effusion (X_3_), changes in the diseased intestinal wall enhancement on enhanced scanning (X_4_), and SBN (Y).

The correlations between four independent variables (X_1_, X_2_, X_3_, and X_4_) and the dependent variable (Y, SBN) were compared between patients in the SBN group and those in the simple SBO group. We used univariate binary logistic regression analysis to identify meaningful variables among the variables (X_1_, X_2_, X_3_, and X_4_), and included them in the multivariate logistic regression analysis. The receiver operating characteristic (ROC) curve was used to calculate the accuracy of the multivariate analysis. The chi-square test and Fisher's exact test were used to test the differences in hospitalization time, time from admission to surgery, and the correlation between age and SBN between patients in the simple group and those in the SBN group.

All the statistical tests in our study were two-tailed, and a *p* value < 0.05 was used to indicate statistical significance. We used SPSS 26 and R 4.3.1 software for statistical analysis.

Cohen's kappa coefficient was used to evaluate the consistency between the two observers. The consistency strength of the kappa coefficient κ was as follows: < 0.2, indicated slight consistency; 0.21–0.40, indicated fair consistency; 0.41–0.60, indicated moderate consistency; 0.61–0.80, indicated substantial consistency; and 0.81–1.00, indicated almost perfect consistency.

## Results

### Patient population

Overall, 186 patients with SBO met all the inclusion and did not meet all exclusion criteria. Patients ranged in age from 2 to 94 years, with a median age of 64.5 years. Seventy-nine patients (42.47%) were female, and 107 patients (57.53%) were male. Among the 186 patients with SBO we selected, 47.3% had SBN. Patients with adhesive SBO were the most common, accounting for 50.00% (93/186) of the patients, adhesive SBO combined with intra-abdominal hernia for 10.75% (20/186), vascular SBO for 8.06% (15/186), intra-abdominal hernia for 7.53% (14/186), external abdominal hernia for 6.99% (13/186), radiation-induced intestinal injury for 4.30% (8/186), SBO caused by fecal stones for 3.76% (7/186) of the patients, small-bowel torsion for 2.69% (5/186), intussusception for 1.08% (2/186), and enteritis or inflammatory bowel disease for 1.08% (2/186). The cause of SBO was unknown, accounting for 3.23% (6/186) of the patients.

The median time from emergency registration to CT examination was 1.2 h (0.5–3.6 h), and the median time from CT examination completion to surgical intervention was 7.3 h (3–12.4 h). Moreover, there was no significant difference in the above time between the simple group and the SBN group (*p* = 0.62). The median hospitalization time was 9 days (4–15 days), and the hospitalization time of patients in the simple group (5.5 days, 4.5–10 days) was significantly lower than that of patients in the SBN group (11.5 days, 10.5–15 days) (*p* < 0.001). Among the 88 patients with SBN, 4 ultimately died of postoperative severe systemic infection and sepsis, while all 98 patients with simple SBO were cured and discharged after surgery.

According to the postoperative pathological examinations, among the 88 patients with SBN, 22 (25.0%) had single-focal SBN, 34 (38.6%) had multifocal SBN, and 32 (36.4%) had diffuse SBN. Seventy-four patients (84.1%) had transmural SBN, and 14 patients (15.9%) had nontransmural SBN. Among the 74 patients with transmural SBN, 11 patients (12.5%, 11/88) had localized or diffuse bleeding in the small-bowel wall. Four patients with reversible small-bowel ischemia were still classified as SBN (4.5%, 4/88).

### Statistical analysis and CT findings

**Fig. 1:**
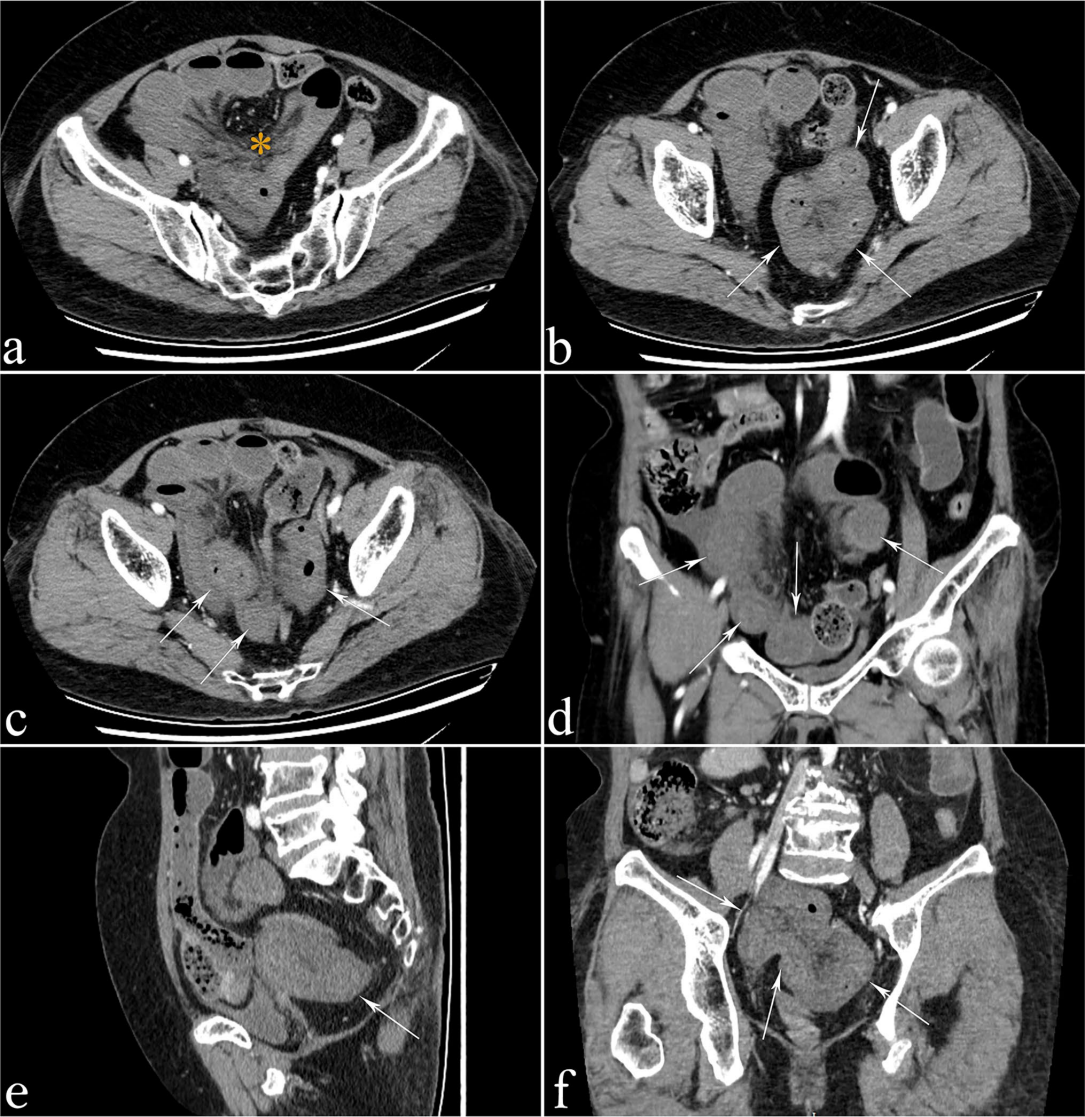
A 59-year-old female patient with adhesive SBO with SBN who underwent appendicitis surgery 2 years ago showed symptoms of abdominal pain and fatigue. **a** The dilated small-bowel indicates SBO, and mesenteric effusion can be seen (*). **b** Diffuse thickening and decreased density of the ileal wall with target signs (arrows) are observed, and the CT value of the small-bowel wall is measured to be approximately 15 HU. **c** After enhanced scanning, the thickening of the ileal wall with the target sign shows a decrease in enhancement (arrow), and the measured CT value of the small-bowel wall is approximately 17 HU, with almost no enhancement. **d** and **e** The CT images with coronal and sagittal reconstructions show a thickened ileal wall with the target sign (arrow). **f** In the image with 3D MPR (Three-dimensional multi-planar reformation), the target sign can be observed (arrow)

**Fig. 2:**
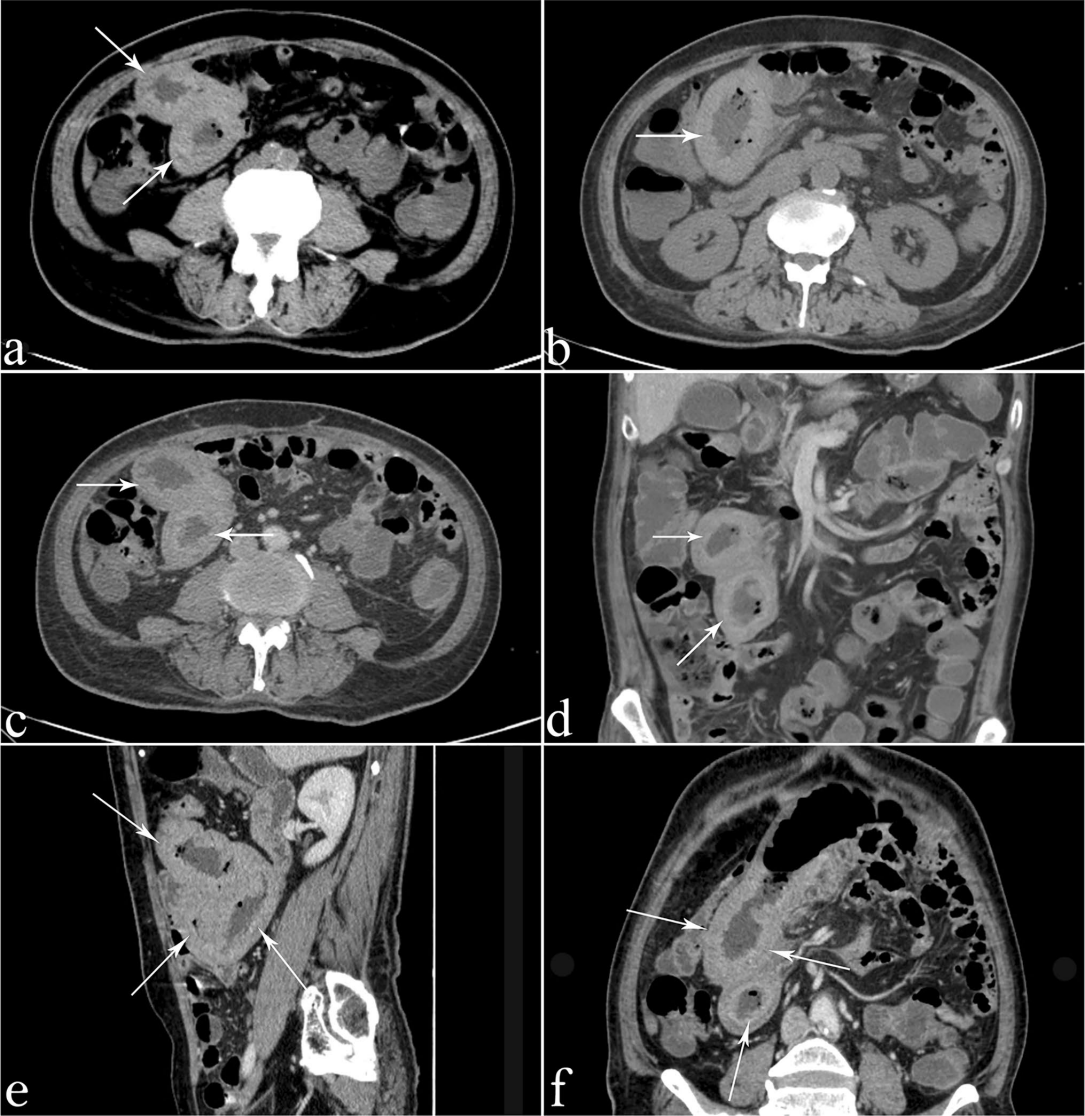
A 46-year-old male patient with adhesive SBO with SBN had undergone appendicitis surgery more than 10 years ago, with symptoms of abdominal pain, nausea, vomiting, and fever. **a** Two thickened and high-density small-bowel walls with the target sign (arrows) can be seen on CT plain scanning, and the CT value of the small-bowel wall on plain scanning is approximately 53 HU. **b** The arrow shows the thickened small-bowel wall with a high-density target sign at different levels. **c** After enhanced scanning, the thickened small-bowel wall with a high-density target sign shows a decrease in enhancement. After enhancement, the CT value of the small-bowel wall is 57 HU, with almost no enhancement. **d** and **e** The coronal and sagittal views show a thickened small-bowel wall with the high-density target sign. **f** The target sign can be seen in the image with 3D MPR (arrow)

**Fig. 3:**
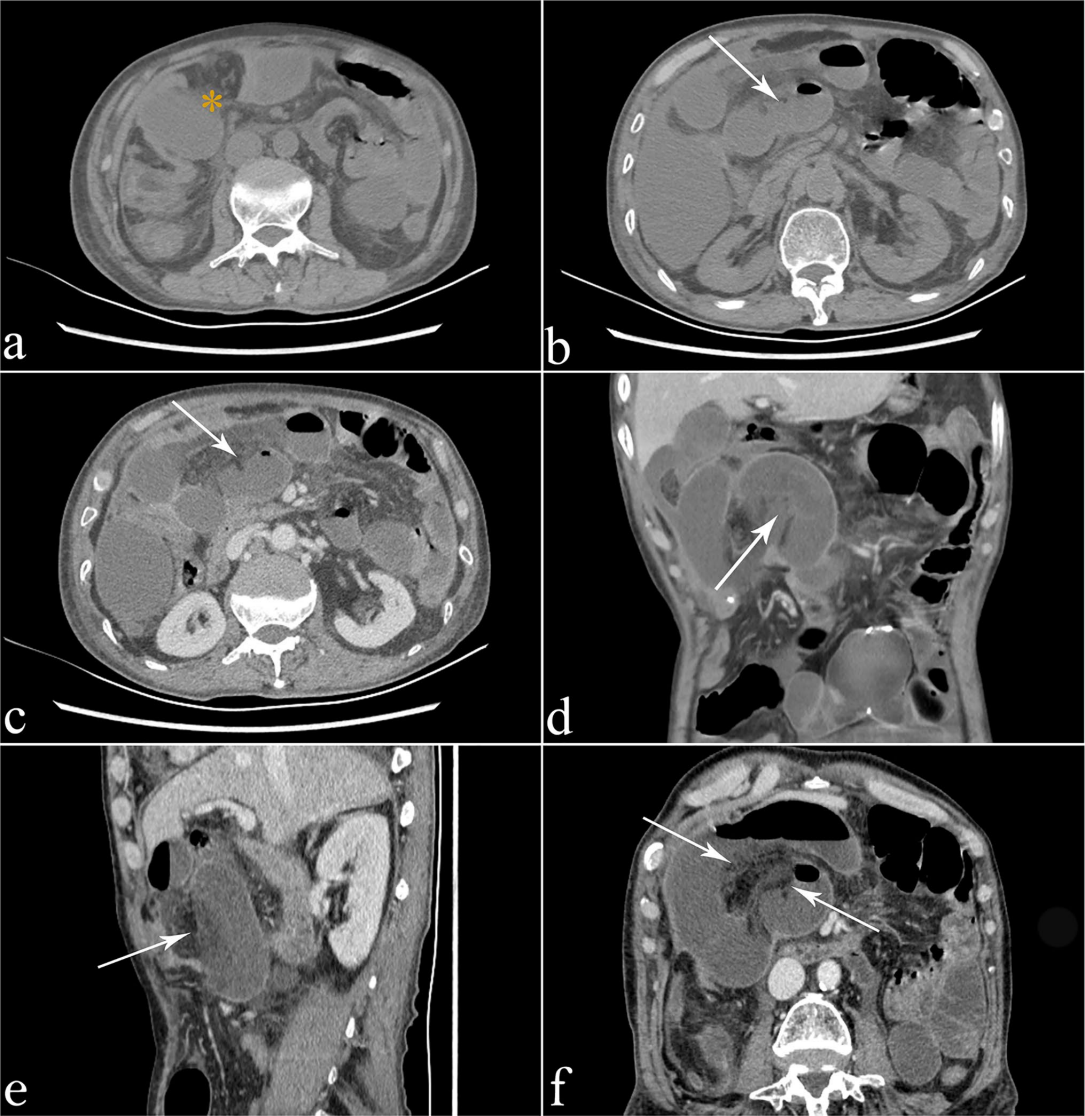
A 65-year-old male patient with vascular SBO with symptoms of abdominal pain, vomiting, and fever. **a** The small-bowel is significantly dilated, accompanied by mesenteric effusion (*), indicating SBO. **b** Partial thinning of the jejunal wall indicates SBN (arrow). **c** After enhanced scanning, the enhancement of the thinner jejunal wall is reduced (arrow). **d** and **e** The coronal and sagittal views show a thinner jejunal wall with lower enhancement (arrow) and surrounding mesenteric effusion. **f** In the image with 3D MPR, the thinner small-bowel wall with surrounding mesenteric effusion indicates SBN (arrow). After communications and researchs with the surgeon, we had determined that the jejunal wall indicated by the arrow was the site of SBN

**Fig. 4:**
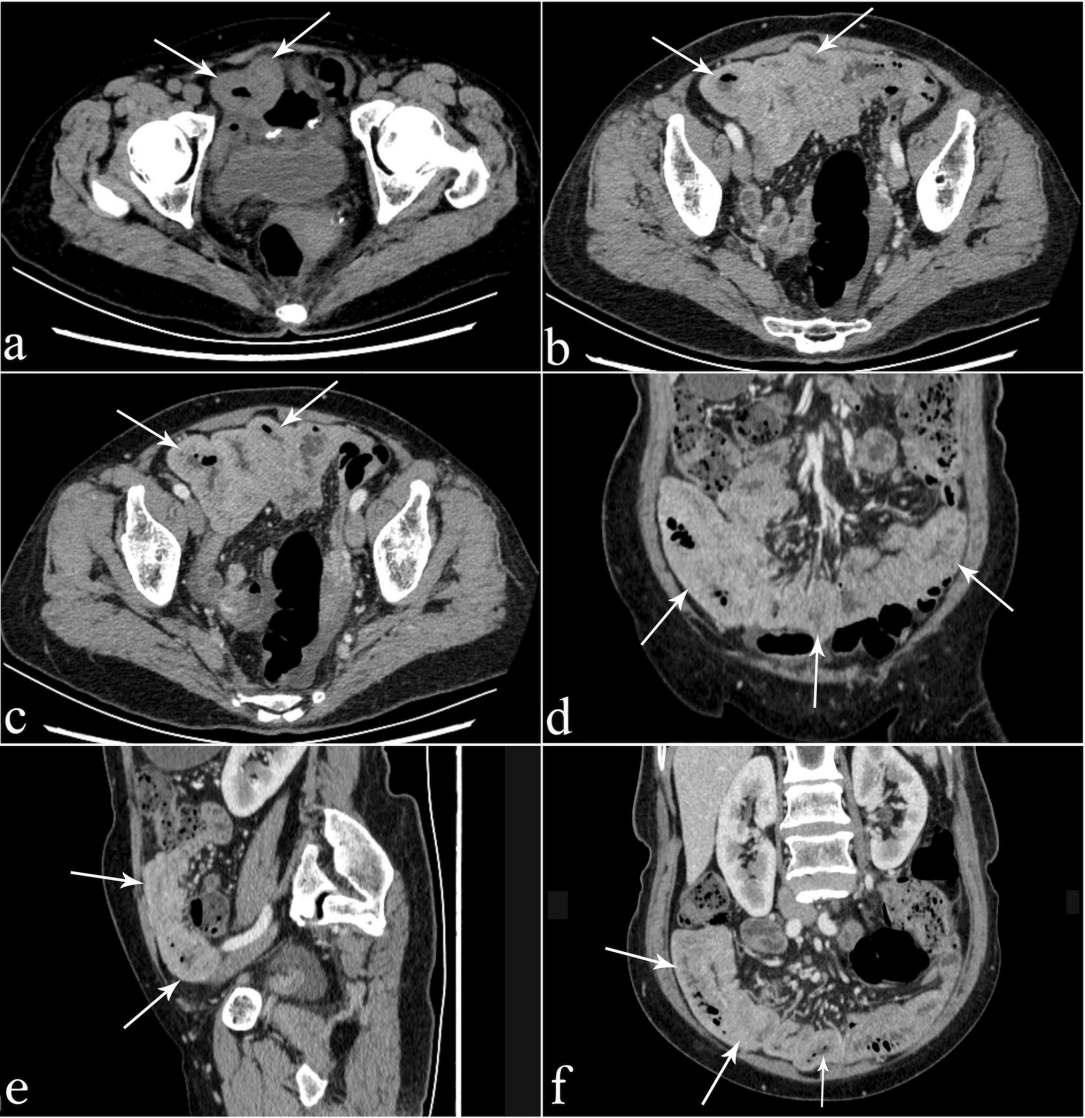
A 66-year-old female patient with adhesive SBO and SBN underwent radical surgery for sigmoid colon cancer 4 years ago, with symptoms of abdominal pain and vomiting. After conservative treatment outside the hospital, small-bowel dilation was slightly alleviated, but symptoms such as abdominal pain did not show significant relief. **a.** Thickened small-bowel wall can be seen in the lower abdomen, presenting as an overall high-density target sign (arrows) on CT plain scanning, and the CT value of the diseased small-bowel wall is approximately 41 HU. **b** and **c** After enhanced scanning, the thickened small-bowel wall with a high-density target sign shows an increase in enhancement. After enhancement, the CT value of the small-bowel wall is 93 HU with significant enhancement, and the CT value of the diseased small-bowel wall increases by approximately 52HU. **d** and **e** The coronal and sagittal views show an thickened small-bowel wall with the overall high-density target sign (arrows). **f** The arrow indicates an overall high-density target sign with significant enhancement in 3D MPR

**Fig. 5:**
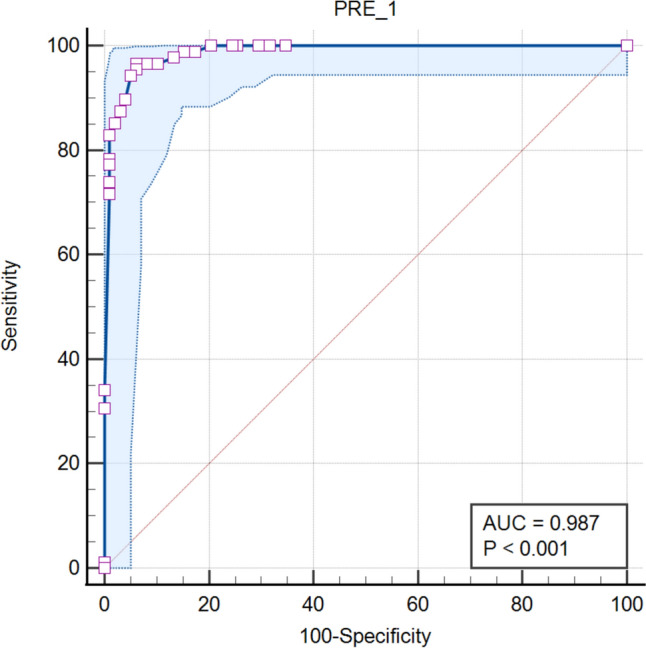
The AUC under the ROC curve in the multivariate analysis was 0.987 (95% CI 0.974–0.999)

According to the univariate analysis, the four variables included in the logistic regression were significantly correlated with SBN, as shown in Table 1. No significant correlation was found between age (*p* = 0.372), sex (*p* = 0.511), and SBN. According to the multivariate logistic regression analysis, 6 CT findings and signs among the four variables were significantly correlated with SBN, and could predict the occurrence of SBN, as shown in Table 2. The 6 CT findings that could predict SBN were as follows: thickening of the diseased intestinal wall with the target sign on plain scanning (OR = 21.615; Figs. 1, 2); thinning of the diseased intestinal wall on plain scanning (OR = 48.106; Fig. 3); increase in the diseased intestinal wall density on plain scanning (OR = 13.696; Fig. 2); and mesenteric effusion (OR = 21.635; Figs. 1, 2); decrease in the diseased intestinal wall enhancement on enhanced scanning (OR = 41.662; Fig. 1); and increase in the diseased intestine wall enhance on enhanced scanning (OR = 15.488; Fig. 4). The ROC curve was used to evaluate the accuracy of the multivariate regression analysis. In our study, the AUC under the ROC curve for the multivariate analysis was 0.987 (95% CI 0.974–0.999) (Fig. 5).

**Table 1:** Univariate regression analysis

CT findings	SBN			OR	95% CI	*p* value
	Yes		No			
Changes in the diseased intestinal wall thickness on plain scanning	No	2/88 (2.27%)	77/98 (78.57%)			< 0.001
	Thickening without the target sign	10/88 (11.36%)	16/98 (16.33%)	24.062	4.806–120.483	< 0.001
	Thinning	31/88 (35.23%)	1/98 (1.02%)	1193.500	104.403–13643.728	< 0.001
	Thickening with the target sign	45/88 (51.14%)	4/98 (4.08%)	433.125	76.269–2469.689	< 0.001
Changes in the diseased intestinal wall density on plain scanning	Normal	7/88 (7.95%)	81/98 (82.65%)			< 0.001
	Reduction	71/88 (80.68%)	8/98 (8.16%)	102.696	35.464–297.384	< 0.001
	Increase	10/88 (11.36%)	9/98 (9.18%)	12.857	3.926–42.104	< 0.001
Mesenteric edema and effusion	Normal	0/88 (0.00%)	30/98 (30.61%)	120.613	38.894–374.033	< 0.001
	Edema	6/88 (6.82%)	61/98 (62.24%)			
	Effusion	82/88 (93.18%)	7/98 (7.14%)			
Changes in the diseased intestinal wall enhancement on enhanced scanning	Normal	4/88 (4.55%)	80/98 (81.63%)			< 0.001
	Reduction	72/88 (81.82%)	7/98 (7.14%)	205.714	57.825–731.831	< 0.001
	Increase	12/88 (13.64%)	11/98 (11.22%)	21.818	5.974–79.680	< 0.001

**Table 2:** Multivariate regression analysis

CT findings	OR	95% CI	*p* value
Thickening of the diseased intestinal wall with the target sign on plain scanning	21.615	1.190–392.609	0.038
Thinning of the diseased intestinal wall on plain scanning	48.106	1.333–1735.846	0.034
Increase in the diseased intestinal wall density on plain scanning	13.696	1.062–176.706	0.045
Mesenteric effusion	21.635	3.063–152.833	0.002
Decrease in the diseased intestinal wall enhancement on enhanced scanning	41.662	3.064–566.427	0.005
Increase in the diseased intestinal wall enhancement on enhanced scanning	15.488	1.103–217.426	0.042

The sensitivity (SEN), specificity (SPE), positive predictive value (PPV) and negative predictive value (NPV) of the 6 CT findings are shown in Table 3. Among the 6 CT findings, the SEN, SPE, PPV, and NPV of “mesenteric effusion” were all greater than 90%. The SPE and PPV of the “thinning of the diseased intestinal wall on plain scanning” were both the highest. However, the recognizability of the above two CT signs was slightly poor, and the recognition of "mesenteric effusion" on CT images was susceptible to interference from hypoproteinaemia or peritonitis, while the recognition of "thinning of the diseased intestinal wall on plain scanning” was easily affected by the significant dilation of the small-bowel. Compared to that of the target sign, the consistency between the interobservations of these two signs was slightly lower, with κ values of 0.72 (95% CI 0.63 ~ 0.79) and 0.59 (95% CI 0.51 ~ 0.74).

**Table 3:** SEN, SPE, PPV, NPV and 95% CI

CT findings	Sensitivity (SEN)	Specificity (SPE)	Positive predictive value (PPV)	Negative predictive value (NPV)
Thickening of the diseased intestinal wall with the target sign on plain scanning	51.14% (40.48–61.79%)	95.92% (91.93–99.91%)	91.84% (83.89–99.78%)	68.61% (60.74–76.48%)
Thinning of the diseased intestinal wall on plain scanning	34.48% (24.29–44.67%)	98.97% (96.92–100%)	96.77% (90.19–100%)	62.75% (55.00–70.49%)
Increase in the diseased intestinal wall density on plain scanning	10.35% (3.82–16.87%)	90.77% (84.84–96.60%)	50.00% (24.41–75.59%)	54.72% (46.90–62.54%)
Mesenteric edema	6.82% (1.45–12.19%)	37.76% (27.99–47.52%)	8.96% (1.94–15.97%)	31.09% (22.65–39.53%)
Mesenteric effusion	93.18% (87.81–98.55%)	92.86% (87.67–98.05%)	92.14% (86.43–97.84%)	93.81% (88.93–98.69%)
Decrease in the diseased intestinal wall enhancement on enhanced scanning	81.82% (73.60–90.04%)	92.86% (87.67–98.05%)	91.14% (84.73–97.55%)	85.05% (78.18–91.91%)
Increase in the diseased intestinal wall enhancement on enhanced scanning	13.64% (6.32–20.95%)	88.78% (82.41–95.14%)	52.17% (30.09–74.26%)	53.37% (45.63–61.11%)

We found that the target sign (thickening of the diseased intestinal wall with the target sign on plain scanning) was a significant CT sign for predicting SBN. Compared to other CT findings, the target sign was easily recognizable on CT images, had a greater consistency between the interobservers (κ = 0.86; 95% CI 0.71 ~ 0.95), and appeared in 49 patients with SBN. This sign had a specificity of 95.92% (SPE; 95% CI 91.93–99.91%), but a slightly lower sensitivity (SEN) of 51.14% (95% CI 40.48–61.79%). The positive predictive value (PPV) was 91.84% (95% CI 83.89–99.78%), and the negative predictive value (NPV) was 68.61% (95% CI 60.74–76.48%). In our study, we also found that the target sign can manifest as both overall low density and overall high density (Figs. 2, 3, 4), with low-density target signs accounting for the majority of the signs, for a ratio of approximately 37:4. After enhanced CT scanning, the majority of patients exhibited a target sign with significantly reduced enhancement, for a ratio of approximately 39:6.

### Consistency between the observers

Among all the CT signs and findings in our study, the CT sign with the best interobserver consistency was “thickening of the diseased intestinal wall with the target sign on plain scanning” (κ = 0.86; 95% CI = 0.71 ~ 0.95), possibly due to the easier recognition of the target sign. The three CT findings showed good interobserver consistency, with κ values ranging from 0.61 to 0.80 among “mesenteric effusion”, “decrease in the diseased intestine wall enhancement on enhanced scanning”, and “increase in the diseased intestine wall density on enhanced scanning”. Among them, “mesenteric effusion” had a relatively better κ value (κ = 0.72; 95% CI = 0.63 ~ 0.79). There were two CT findings with slightly lower consistency between the interobservers, namely, “thinning of the diseased intestine wall on plain scanning” (κ = 0.59; 95% CI = 0.51 ~ 0.74) and “increase in the diseased intestine wall density on plain scanning” (κ = 0.60; 95% CI = 0.53–0.72), which may be related to the difficulty in accurately measuring changes in diseased intestinal wall thickness and density due to the intestinal dilation in patients with SBO.

## Discussion

In our study, we aimed to investigate the possible changes in the thickness and density of diseased intestinal walls in SBO patients with SBN and to provide valuable insights into how to predict SBN from the perspective of possible abnormal changes in the diseased intestinal wall itself. Therefore, we did not include additional clinical or laboratory indicators, nor did we include other CT signs, such as the fecal sign, the whirl sign, or small-bowel wall gas accumulation, because the occurrence of these CT signs and abnormalities in clinical indicators may not be universal and may not occur in some patients with SBN.

Multivariate regression analysis revealed that 6 CT findings were significantly correlated with SBN: thickening of the diseased intestinal wall with the target sign on plain scanning (OR = 21.615), thinning of the diseased intestinal wall on plain scanning (OR = 48.106), increase in the diseased intestinal wall density on plain scanning (OR = 13.696), mesenteric effusion (OR = 21.635), decrease in the diseased intestinal wall enhancement on enhanced scanning (OR = 41.662), and increase in the diseased intestinal wall enhancement on enhanced scanning (OR = 15.488). The area under the ROC curve (AUC) of the six CT findings in the multivariate analysis was 0.987 (95% CI 0.974–0.999; Fig. 5). Specifically, the specificity (SPE, 95.92%; 95% CI 91.93–99.91%) and positive predictive value (PPV, 91.84%; 95% CI 83.89–99.78%) of the target sign were both high and the target sign was easy to recognize on CT images, suggesting that the target sign is a significant CT sign for predicting SBN. When this CT sign is observed, it may be helpful in distinguishing simple SBO from SBN.

In our study, the patients had a relatively high incidence of SBN, reaching 47.3%, which might be mainly due to the underdeveloped economy in this region and the delay of treatment for patients with SBO.

Several studies have shown that the small-bowel fecal sign is a protective factor against SBN [18, 19]. Other studies have shown that the whirl sign is mainly observed in patients with small-bowel torsion or adhesive small-bowel obstruction with small-bowel torsion and may predict SBN [20, 21]. Research by Paul Leber and his colleagues showed that the small-bowel wall pneumatosis sign and portal vein pneumatosis sign were not specific for predicting SBN [22].

Mahdi Bouassida et al. developed a predictive model and identified six independent predictive factors for SBN, including age, duration of pain before admission, body temperature, WBC, reduced enhancement of the intestinal wall on CT scans, and mesenteric effusion on CT scans [23]. This model focuses more on the predictive role of clinical findings. In our study, there was no significant correlation between age and SBN. After enhanced CT scanning, some patients with SBN may have greater intestinal wall enhancement than normal patients.

Kazuaki Nakashima et al. studied the CT findings of closed-loop SBO and focused more on mechanical intestinal obstruction [24]. However, whether this study is applicable to other types of intestinal obstruction, such as vascular intestinal obstruction, remains to be further validated. In their study, it was shown that decreased enhancement of the diseased intestinal wall was an important finding in predicting SBN, which was not entirely consistent with our study. Our study revealed that when SBN occurred, a certain proportion of patients exhibited significantly increased enhancement of the diseased intestinal wall.

Camille Rondenet et al.'s research on closed-loop SBO showed that when patients with SBO had SBN, only CT findings of an increased intestinal wall density higher than normal had predictive value for SBN [5]. In our study, we found that a significant increase in the density of the diseased intestinal wall was indeed an important CT finding, but it was not the only one. Five other CT findings could help predict SBN. Their research also focused on mechanical intestinal obstruction, but it was unclear whether this approach was applicable to other types of SBO.

Research by Zhenkai Li and his colleagues showed that the “fish tooth sign”, “bowel wall thickening”, and “mesenteric edema” could predict SBN on CT images [25]. However, our study showed that simple thickening of the intestinal wall was not significantly correlated with the SBN according to multivariate regression analysis. Only “thickening of the diseased intestinal wall with the target sign” was a meaningful factor in predicting SBN.

A study by Shannon P Sheedy et al. showed that a significant decrease in the enhancement of the diseased intestinal wall on enhanced CT was an independent predictor of SBN, which was not fully consistent with our study [26]. This may be due to the small sample size of their study (n = 61). Our study suggested that a significant increase in diseased intestinal wall enhancement may also occur in patients with SBN (Fig. 4).

Compared to those in other studies, the patients included in our study not only had the most common mechanical intestinal obstruction but also had vascular intestinal obstruction, as well as intestinal obstruction caused by radiation-related intestinal injury and inflammatory intestinal disease. Therefore, the conclusions drawn from our study may have wider applicability.

However, our research has three limitations. First, our study included only patients with SBO who received surgical treatment at our hospital, while those who did not receive surgical treatment were not included in our study. Moreover, this was a retrospective study, which may have lead to selection bias. Second, due to the significant dilation of the small-bowel lumen in some patients with SBO, the thickness and density of the intestinal wall on CT images could not be quantitatively and accurately measured using the region of interest (ROI) due to the small ROI area, and could only be evaluated using subjective vision. Although we used Cohen's kappa coefficient to evaluate the consistency between interobserver distributions, deviations were inevitable. Finally, our study was a single-center study, and further validation of our conclusions is needed by including additional patients from multiple centers.

## Conclusion

In summary, we identified 6 CT findings that were significantly correlated with SBN. Compared to other CT features, these 6 CT findings reveal changes in thickness and density that may occur in the ischemic and necrotic small-bowel wall itself, increasing the universality and significance of the findings in predicting SBN. Among these signs, the target sign is easier to recognize and has high specificity and positive predictive value, making it a significant CT finding for predicting SBN, and it has important implications for clinical treatment.

## Data Availability

All the data supporting the conclusions of this study are included in this article. For further requests, please contact the corresponding author.

## Abbreviations

ROC: Receiver operating characteristic
AUC: Area under the receiver operating characteristic curve
CI: Confidence interval
CT: Computed tomography
HU: Hounsfield units
SBO: Small-bowel obstruction
SBN: Small-bowel ischemia and necrosis
OR: Odds ratio
ROI: Region of interest
SPE: Specificity
SEN: Sensitivity
PPV: Positive predictive value
NPV: Negative predictive value
3D MPR: Three-dimensional multi-planar reformation

## References

[1] Scaglione M, Galluzzo M, Santucci D (2022). Small bowel obstruction and intestinal ischemia: emphasizing the role of MDCT in the management decision process. Abdom Radiol.

[2] Kim HR, Lee Y, Kim J (2023). Closed loop obstruction of small bowel: CT signs predicting successful non-surgical treatment. Eur J Radiol.

[3] Xu WX, Zhong QH, Cai Y (2022). Prediction and management of strangulated bowel obstruction: a multi-dimensional model analysis. BMC Gastroenterol.

[4] Santillan CS (2013). Computed tomography of small bowel obstruction. Radiol Clin N Am.

[5] Millet I, Taourel P, Ruyer A (2015). Value of CT findings to predict surgical ischemia in small bowel obstruction: a systematic review and meta-analysis. Eur Radiol.

[6] Davarpanah AH, Ghamari KA, Khosravi B (2021). Many faces of acute bowel ischemia: overview of radiologic staging. Insights Imaging.

[7] Ozawa M, Ishibe A, Suwa Y (2021). A novel discriminant formula for the prompt diagnosis of strangulated bowel obstruction. Surg Today.

[8] Podda M, Khan M, Di Saverio S (2021). Adhesive small bowel obstruction and the six w's: Who, how, why, when, what, and where to diagnose and operate?. Scand J Surg.

[9] Zielinski MD, Eiken PW, Heller SF (2011). Prospective, observational validation of a multivariate small-bowel obstruction model to predict the need for operative intervention. J Am Coll Surgeons.

[10] Kobayashi T, Chiba N, Koganezawa I (2022). Prediction model for irreversible intestinal ischemia in strangulated bowel obstruction. BMC Surg.

[11] HUang X, Fang G, Lin J (2018). A prediction model for recognizing strangulated small bowel obstruction. Gastroent Res Pract.

[12] Yu H, Kirkpatrick I (2023). An update on acute mesenteric ischemia. Can Assoc Radiol J.

[13] Diamond M, Lee J, LeBedis CA (2019). Small bowel obstruction and ischemia. Radiol Clin N Am.

[14] Idelevich E, Kashtan H, Mavor E (2006). Small bowel obstruction caused by secondary tumors. Surg Oncol.

[15] Olson MC, Navin PJ, Welle CL (2021). Small bowel radiology. Curr Opin Gastroen.

[16] Mallo RD, Salem L, Lalani T (2005). Computed tomography diagnosis of ischemia and complete obstruction in small bowel obstruction: a systematic review. J Gastrointest Surg.

[17] Hines J, Rosenblat J, Duncan DR (2013). Perforation of the mesenteric small bowel: etiologies and CT findings. Emerg Radiol.

[18] Yamamoto Y, Miyagawa Y, Kitazawa M (2021). Association of feces sign with prognosis of non-emergency adhesive small bowel obstruction. Asian J Surg.

[19] Berl S, Dawkins A, DiSantis D (2016). The small bowel feces sign. Abdom Radiol.

[20] Duda JB, Bhatt S, Dogra VS (2008). Utility of CT whirl sign in guiding management of small-bowel obstruction. Am J Roentgenol.

[21] Barberi C, Colaizzi C, Guerrini J (2021). Whirl sign: a common misinterpreted radiological entity. Intern Emerg Med.

[22] Lebert P, Ernst O, Zins M (2021). Pneumatosis intestinalis and portal venous gas in mechanical small bowel obstruction: Is it worrisome?. Diagn Interv Imaging.

[23] Bouassida M, Laamiri G, Zribi S (2020). Predicting intestinal ischaemia in patients with adhesive small bowel obstruction: a simple score. World J Surg.

[24] Nakashima K, Ishimaru H, Fujimoto T (2015). Diagnostic performance of CT findings for bowel ischemia and necrosis in closed-loop small-bowel obstruction. Abdom Imaging.

[25] Li Z, Shi L, Zhang J (2022). Imaging Signs for Determining Surgery Timing of Acute Intestinal Obstruction. Contrast Media Mol I.

[26] Sheedy SP, Earnest FT, Fletcher JG (2006). CT of small-bowel ischemia associated with obstruction in emergency department patients: diagnostic performance evaluation. Radiology.

